# The Therapeutic Uses of Cocaine

**Published:** 1887-08

**Authors:** 


### THE THERAPEUTIC USES OF COCAINE.

THE value of coca preparations for internal administration is so positively assured by a large number of definite experiments in different clinics, that the practitioner can no longer refuse to admit the important service to be derived from it when given in response to definite indications. As an analeptic or restorative agent, it has been long known, whilst most recently it has been recognized as an excellent stomachic tonic, and has, for example, been recommended by Professor Pott, of Halle, as a remedy for the enervating diarrhœa of children. He claims the most brilliant results from the use of the alcoholic tincture of coca leaves for this purpose. Of much greater significance, however, seems to be the therapeutic effect of cocaine as an antispasmodic, a fact that has been established by Pott and others in cases of epilepsy, eclampsia, spasm of the glottis, pertussis and chorea. It therefore seems opportune to add to the results some further observations of the successful application of cocaine in the practice of the writer to cases of whooping cough, neuralgia of the stomach, and bronchial asthma.

### I. COCAINE IN CASES OF WHOOPING COUGH.

During the past summer I made the experiment of the application of cocaine in twelve cases of whooping cough, in children from 1 to 7 years of age, that had been sent to the Sanitary Institution of Colberg. At first I tried the commonly recommended practice of touching, several times a day, the posterior wall of the pharynx and the epiglottis with a 10 per cent. solution of the drug. But no good effects at all followed this; indeed, the mechanical irritation of the brush produced, on the contrary, the most violent paroxysms of coughing on the part of the struggling little patients.

I therefore ordered instead a one per cent. solution (gr. iss. to ℥iiss. aquæ amygdal. am.) for internal administration, giving the child, according to its age, gtt. 10–15–20, three to four times daily in a lump of hard sugar, with the object of keeping the cocaine for a longer time in intimate contact with the pharyngeal walls by chewing than by simply swallowing the liquid. On an average, within three days the attacks became much less severe, the nights became more peaceful, the debilitating emesis ceased, and in two to three weeks the persistence of a slight catarrh was all that remained. The children, in the fresh sea-breezes and with a few salt-baths, quickly regained the most perfect health. Although it is well-known that a change of air, especially a stay at the sea-shore, operates most happily in relieving whooping cough, the author emphasizes his eonviction that during no previous summer has he seen the acute stage of the malady so quickly and easily passed through as during the last season, when he adopted the use of cocaine as above described.

II. COCAINE IN GASTRIC NEURALGIA.

The author also reports the very favorable action of the internal administration of the hydrochlorate of cocaine in three cases of severe neuralgia of the stomach in young women of 20, 22, and 26 years, in whom there was neither pronounced anæmia, suspicion of gastric ulcer, nor disorder of the genital functions. It was best borne when conbined with belladonna in pill form:

℞. Cocain. hydrochlor. . gr. iss.
Extr. Belladon. . . . . . gr. viiss.
Extr. Rh. . . . . . . . . . . q. s.
To make 20 pills.
Sig. One pill after meals three times daily.

The first two cases were two sisters of Warschau. They consulted me in August of the previous year because of seizures of violent gastralgia immediately after every surf-bath, although they had suffered from lighter attacks at times before this. Both of the girls, as I have said, had the appearance of enjoying perfect health; there was no trace of anæmia, and all functions in the best order; palpitation of the stomach showed no sensitive spots. Several days' use of the cocaine pills gave decided relief. The same formula was used later at their home during the winter, with the same happy results.

In the third patient the gastralgia was combined with hiccough that came on during the night and was extremely tormenting. In this case, it is true, I was unable to put aside entirely the suspicion of hysteria, although the lady seemed in every respect to be healthy and strong. Cocaine in this instance produced its anti-spasmodic effects. Two pills were given at night before going to bed, with the immediate result of bringing better sleep. This, with sea-bathing, finally affected a complete cure.

III. COCAINE INHALATION IN BRONCHIAL ASTHMA.

I have only one case of bronchial asthma to report, but that one was very accurately studied by me, and cocaine, by inhalation, was used with the most excellent results. To my knowledge it has not previously been used for these indications in the same form. The patient was a solidly-built woman of fifty years, from the neighborhood of Colberg. In November of the previous year, after a severe cold, she was one night suddenly seized with what seemed from all appearances to be a laryngeal croup. I found her nearly stifling. Breathing was terribly difficult, all the accessory muscles of respiration in extreme activity, the face livid, the extremities covered with cold sweat, the pulse small and excessively rapid. A powerful emetic fortunately succeeded in speedily bringing out of the larynx and trachea a large mass of tough, fibrous membrane, giving her speedy relief. Succeeding this croupous attack followed an obstinate bronchial catarrh, lasting several weeks, resulting in frequent and severe asthmatic seizures. Morphine, internally or subcutaneously, soon ceased to afford relief, so that I hit upon the happy thought of trying the inhalation of cocaine. I found it, in fact, the best remedy, and I do not hesitate most readily to advise its use in like circumstances, when morphine is contraindicated, or has failed to produce the desired effect.

In my case I allowed the patient to inhale about ℥v. of a one per cent. solution, by means of one of our common inhalation apparatuses, requiring about five minutes' time when the machine is properly in action. This amount was entirely sufficient the first time to cut short a severe attack. Subsequently the patient learned to begin the inhalation at the commencement of the attack, and then ten or twenty inhalations were sufficient to give instantaneous relief.

Besides, it must be remembered that one cannot inhale much more than this amount without superinducing the known effects of the drug, the contraction of the vessels of the mucous membrane, the extreme resultant dryness, and an uncomfortable feeling, ending in producing a sensation of tickling in the throat, whilst at the same time, lips, tongue and tonsils show the greatest paleness and insensibility.—*Dr. Weissenberg, in the Allgem. Med. Cent.-Zeitung.*

